# Development of Synergy-Based Combination for Learning and Memory Using *in vitro*, *in vivo* and TLC-MS-Bioautographic Studies

**DOI:** 10.3389/fphar.2021.678611

**Published:** 2021-07-02

**Authors:** Maaz Ahmed Khan, Varsha Srivastava, Mariya Kabir, Monalisha Samal, Areeba Insaf, Mohammad Ibrahim, Sultan Zahiruddin, Sayeed Ahmad

**Affiliations:** Bioactive Natural Product Laboratory, Department of Pharmacognosy and Phytochemistry, School of Pharmaceutical Education and Research, Jamia Hamdard, New Delhi, India

**Keywords:** *Withania somnifera*, *Myristica fragrans*, synergy, anticholinesterase, neurodegenerative, TLC-bioautography

## Abstract

The present study is aimed at developing a synergistic combination to enhance learning and memory in Alzheimer’s patients with the help of eight common medicinal plants used in the AYUSH system. Aqueous and hydroalcoholic extracts of eight medicinal plants from the AYUSH system of medicine were prepared. These were subjected to *in vitro* anticholinesterase activity, to find out the combination index of synergistic combination. The synergistic combination and their individual extracts were subjected to total phenol, flavonoid and antioxidant activity estimation. Further, *in vivo* neurobehavioral studies in rats were carried out followed by TLC-MS-bioautographic identification of bioactive metabolites. Out of the sixteen extracts, aqueous extracts of *Withania somnifera* (L.) Dunal (WSA) and *Myristica fragrans* (L.) Dunal (MFA) were selected for the development of synergistic combination based on their IC_50_ value in *vitro* anticholinesterase assay. The synergistic combination inhibited the anticholinesterase activity significantly as compared to the individual extracts of WSA and MFA. The synergistic combination also showed more phenolic and flavonoid contents with potential antioxidant activity. The TLC-bioautography showed four white spots in WSA, signifying sitoindosides VII, VIII, quercetin, isopelletierine and Withanolide S as AChE inhibitory compounds while showing five white spots of anti-cholinesterase active metabolites identified as eugenol, methyl eugenol, myristic acid, galbacin and β-sitosterol in MFA. The observation of neurocognitive behavior in amnesia induced subjects manifested that both the synergistic combinations showed comparable results to that of standard piracetam, though the synergistic combination containing a higher concentration of WSA showed more appreciable results in ameliorating dementia in rats. The study suggests that the synergy based combination successfully enhanced memory and learning by abating free radical and acetylcholine levels, and increased learning and memory in rats, providing a strong rationale for its use in the treatment of dementia and Alzheimer’s disease.

## Introduction

The cognitive ability is hampered due to factors such as chemicals, genetic relationships, medicaments, disorders and ageing further contributing to the impairment of learning ability. Cognitive impairment may result in dementia or Alzheimer’s disease (AD) (Benson et al., 2014).

Decline in cognitive ability leads to loss of memory i.e., dementia, which leads to imbalance inoccupational as well as social life. The loss of cognitive abilities must be present in several cognitive domains (often memory with at least one other domain such as language, visuo-spatial or executive (Puttarak et al., 2017). Although dementia is common in very elderly people, it is not part of normal ageing (Zahiruddin et al., 2020a). Of all the causes, declining age is the major factor contributing to all-cause dementia. AD affects 5–10% of people older than 65 years and 50% of those aged 85 years (Balkrishna and Misra, 2018). The report of Alzheimer’s Disease International (ADI) (2015) documents that each year more than 9.9 million new cases of dementia erupt worldwide (One new case every 3.2 s) (Prince et al., 2015). Dementia is a common public health problem (Ven Murthy et al., 2012). Worldwide, approximately 47 million people have dementia, and this number is expected to increase to 131 million by 2050 (Ven Murthy et al., 2012). The aetiology of this disease is very complex and is characterized by symptoms that include memory and language impairment, cognitive dysfunction, and behavioral disturbances (i.e., depression, agitation and psychosis) (Prince et al., 2015).

AD is characterized by selective neuronal cell death, the presence of extracellular amyloid deposits in the core of neuritic plaques and the formation of intraneuronal neurofibrillary tangles in the brain of afflicted individuals. When observed through neurochemical aspect, these occur due to inability of cortically projecting cholinergic neurons and the depression of presynaptic markers of the sympathomimetic system specifically in the areas of brain associated with memory and learning ability (Iqbal and Grundke-Iqbal, 2008; Madhusoodanan, 2014). Acetylcholinesterase (AChE) inhibitors, including rivastigmine, tacrine, donepezil, and galanthamine are the most prevalent pharmacological therapies for AD. However, short half-lives and severe side effects such as hepatotoxicity, which is the more frequent side effect of these therapies exist as limitations (Iqbal and Grundke-Iqbal, 2008). These therapies address only symptoms but not disease progression.

AD is associated with several pathologies, including oxidative stress, inflammation, and hyperhomocysteinemia; therefore, a multi-targeted approach may be essential to effectively treat this condition. Drugs derived from the traditional system of medicine have been in practice at a global level for the prophylaxis and treatment of disorders associated with neurobehavioral system and can be a stepping stone for safe and effecacious multi-targeted therapies for the cure of complex neurobehavioral disorders such as AD (Bores et al., 1996).

India is a vast repository of medicinal plants that are used in traditional medical treatments. The rich source of Indian medicinal plants and its applications are well documented in indigenous systems as in Ayurveda, Unani and Siddha (Vuorela et al., 2012). In the modern era there are various plants stated in the Indian System of Medicine that show their cognition-enhancing ability and evidence of anticholinesterase activity such as *Bacopa monnieri* (L.) Wettst. (Das et al., 2002), *Piper nigrum* L. (Ingkaninan et al., 2003), *Withania somnifera* (L.) Dunal (Kurapati et al., 2013), *Centella asiatica* (Linn.) Urban (Mukherjee et al., 2007), *Nardostachys jatamansi* (D.Don) DC (Mukherjee et al., 2007), *Myristica fragrans* Houtt. (Mukherjee et al., 2007), *Tinospora cordifolia* (Willd.) Miers. (Vinutha et al., 2006) and *Butea monosperma* (Lam.) Kuntze (Ingkaninan et al., 2003).

The present study was conducted to develop a synergy based combination for learning and memory enhancement from medicinal plants of AYUSH, having anti-cholinesterase potential using *in vitro* studies, combination index analysis, metabolomics and TLC-MS-bioautography followed by *in vivo* studies. Selected Indian medicinal plant materials are known to be a good source of phenolic, flavonoid and antioxidant compounds, and might contribute to anticholinesterase activity. TLC-MS-bioautography, a technique that integrates bioactivity detection and chromatographic analysis has been used by various scientists across the globe for identification of biologically active compounds with varied specificity and potential therapeutic effect (Goodall and Levi, 1946). Incorporation of TLC-bioautography hyphenated with mass spectrometry (MS) in the study was important for screening and isolation of compounds with potent AChE inhibitory activity owing to its simple, quick, efficient and economical technique.

## Materials and Methods

### Procurement of Plants and Chemicals

Different parts of plants were procured from the herbal drugs market (Universal Trading Company) in Delhi, India (Table 1), DTNB (5, 5-dithiobis-(2- nitrobenzoic acid)); bovine serum albumin, AChE, acetylthiocholine iodide, 2, 2-diphenyl-1-picrylhydrazyl (DPPH), gallic acid, rutin, withanolide S and myristicin were procured from Sigma-Aldrich, United States. Other chemicals and reagents used were of analytical grade (AR) and procured from SRL chemicals, India.

**TABLE 1:** Procurement of selected plants and their voucher specimen number.

S.N.	Botanical name of the plants	Family	Plant part	References	Voucher number
1	*Bacopa monnieri* (L.) Wettst	Scrophulariaceae	Whole	Anonymous (1999)	BNPL/JH/MPH/MAK-18–01
2	*Piper nigrum* L	Piperaceae	Fruits	Anonymous (2001a)	BNPL/JH/MPH/MAK-18–02
3	*Withania somnifera* (L.) Dunal	Solanaceae	Roots	Anonymous (2001b)	BNPL/JH/MPH/MAK-18–03
4	*Centella asiatica* (Linn.) Urb	Umbelliferae	Whole	Anonymous (2004)	BNPL/JH/MPH/MAK-18–04
5	*Nardostachys jatamansi* (D.Don) DC.	Valerianaceae	Rhizomes	Anonymous (2001c)	BNPL/JH/MPH/MAK-18–05
6	*Myristica fragrans* Houtt	Myristicaceae	Aril	Anonymous (2009)	BNPL/JH/MPH/MAK-18–06
7	*Tinospora cordifolia* (Willd.) Miers	Menispermaceae	Stem	Anonymous (2001d)	BNPL/JH/MPH/MAK-18–07
8	*Butea monosperma* (Lam.) Kuntze	Leguminosae	Stem bark	Anonymous (2006)	BNPL/JH/MPH/MAK-18–08

### Preparation of Extracts

Powdered plant materials were macerated separately in aqueous (A) and 50% alcohol (hydroalcoholic, HA) for 24 h. Further, same were extracted using a reflux assembly for 3 h. The extract was concentrated on a water bath at 50°C to form a solid sticky mass. Thereby, % extractive yield for each extract was calculated and the prepared extract was stored at 4°C in a suitable container for further use.

### *In vitro* Anticholinesterase Studies

The estimation of AChE activity was done using Ellman’s spectrophotometric method (Ellman et al., 1961). Acetyl thiocholine iodide (ATCI) acted as a substrate for the reaction along with the utilization of electric eel AChE. Further 5, 5-dithiobis (2-nitrobenzoic) acid (DTNB) was used for the measurement of AChE activity. The hydrolysis of substrate in the presence of the enzyme resulted in the formation of thiocholine as a product which further reacted with Ellman’s reagent (DTNB) to produce 2-nitrobenzoate-5-mercaptothiocholine and 5-thio-2- nitrobenzoate and was detected at 412 nm. The hydrolysis of the ATCI was measured by the formation of the coloured product 5-thio-2-nitrobenzoate anion formed by the reaction of DTNB and thiocholine, which was released by the hydrolysis of the enzyme. The formation of the coloured product was measured at 410 nm wavelength after 10 min. Galanthamine, a standard AChE inhibitor, was used as positive control, which was dissolved in ethanol. Percent AChE inhibition was calculated using the following formula.

Percentage inhibition= 100 - [(Absorbance of the test compound/Absorbance of the solvent)∗100]

The experiment was performed in triplicate and linear regression analysis was done to determine the concentrations of the test extract responsible for inhibiting the hydrolysis of the substrate (acetylcholine) by 50% (IC_50_) by plotting a graph between the inhibition percentages vs. the extract concentration.

The combinations of extracts were screened based on their IC_50_ values out of which two best active combinations were selected. These two combinations were further evaluated to find out the best combination by combination index. Both the extracts were simultaneously treated with the enzyme as selected for each combination using the method of constant ratio drug combination (CHOU and TALALAY, 1981; Chou et al., 1994). The formula for combination index (CI) is as follows:

CI = (dA/DA) + (dB/Db)

Whereas, dA and dB are the concentration of drug A and B used in combination to achieve x% drug effect. DA and DB are the concentrations for single agents to achieve the same effect.

If the combination index values were <1, the combination showed synergism, while value equal to 1 showed additive effect. If the values were >1, the combination showed antagonism (CHOU and TALALAY, 1981; Chou et al., 1994).

### Determination of Total Phenolic Content

Determination of total phenolic content was performed by using the Folin Ciocalteu reagent method (Gaurav et al., 2020) with slight modification. Initially stock solution (5 mg/ml) was prepared from each extract and combination. An amount of 0.2 ml of the test sample was mixed with 0.2 ml of Folin-Ciocalteu’s phenol reagent and 0.6 ml of water. After 5 min, 1 ml of saturated sodium carbonate solution (8% w/v) was added into the mixture. The volume was made up to 3 ml with distilled water. The reaction was allowed to proceed in the dark for 30 min. The obtained sample was further centrifuged and the absorbance of the samples was noted down at 765 nm. The phenolic content was calculated as mg gallic acid equivalents per Gram (mg GAE/g) of dried plant material based on the standard curve of gallic acid (20–100 μg/ml).

### Determination of Total Flavonoid Content

Total flavonoid content of the sample was determined by the aluminium chloride method (Gaurav et al., 2020). Initially stock solution (5 mg/ml) was prepared from each extract and combination. An amount of 0.6 ml of the test samples were separately mixed with 0.6 ml of 2% aluminium chloride. After mixing, the solution was kept at incubation for 60 min at room temperature. UV-Vis spectrophotometer was used at a wavelength of 420 nm for the measurement of absorbance of the reaction mixtures against blank. The concentration of total flavonoid content in the test samples were calculated from the calibration plot of rutin (20–100 μg/ml) and expressed as mg Rutin equivalent per Gram (mg RE/g) of dried plant material.

### *In vitro* Antioxidant Activity

#### DPPH Assay

DPPH (1, 1-diphenyl-2-picryl-hydrazyl) assay method reported (Shen et al., 2010) was conducted to determine the potential of extracts against free radicals. DPPH being a free radical undergoes reduction and accepts hydrogen radical or an electron to become a stable diamagnetic molecule when it reacts with an antioxidant compound that can donate hydrogen and gets reduced. The antioxidant potential was determined by observing the intensity of colour change from deep violet to light yellow which is directly proportional to the nature and amount of free radical scavenging species (antioxidant compounds) present in the sample.

Briefly, a 0.1 mM solution of DPPH in methanol was prepared, and 280 μL of DPPH solution was added to 20 μL of the test solution at different concentration (10, 50, 100, 500, and 1,000 μg/ml) in each microplate well. The mixtures were allowed to stand at room temperature for 30 min in a dark place. Then the plate was shaken for 10 s in a microplate reader and the absorbance was recorded at 517 nm. Ascorbic acid was used as the reference. The free radical scavenging potential is inversely proportional to the absorbance value. Lower the absorbance value of reaction mixture, higher the antioxidant potential and vice versa. Antioxidant activity (free radical scavenging potential) can be inferred by using the following formula.

DPPH scavenging effect (% inhibition) = {((A0-A1)/A0)∗100}

Where A0: absorbance of the control reaction and A1: absorbance in presence of extract samples and reference. The experiment was performed in triplicate and the average was calculated.

#### Reducing Power Assay

The reducing power of selected two extracts was determined by a slight modification of the reported method (Oyaizu, 1986). An amount of 1 ml of the test samples were mixed thoroughly with 2.5 ml of potassium ferricyanide, and 2.5 ml of phosphate buffer and the mixture were kept at 50°C in a water bath for 20 min. After cooling, 2.5 ml of 10% trichloroacetic acid was added and centrifuged at 3,000 rpm for 10 min. The upper layer of solution (2.5 ml) was combined with distilled water (2.5 ml) and a freshly prepared ferric chloride solution (0.5 ml). The absorbance was noted at 700 nm. Control was prepared similarly excluding samples. Ascorbic acid was used as a standard. The reducing power is directly proportional to absorbance i.e., higher the absorbance, higher the reducing power and vice versa.

### TLC Fingerprinting and Chemical Analysis of Selected Extracts

The two selected best active extracts were dissolved in methanol (30 mg/ml) and solutions were filtered through a 0.25µ membrane filter and stored in a container for TLC analysis. For TLC analysis, 5 µL prepared samples from WSA and MFA were separately applied through CAMAG Linomat V automatic sample spotter (CAMAG Muttenz, Switzerland) on silica gel 60 F254 precoated TLC aluminium plates (5 cm × 10 cm), with the help of CAMAG Linomat-V applicator on 6.0 mm wide band and a flow of sample application was set at 120 nL/s. The plates were eluted to a distance of 8.0 cm at room temperature in the mobile phase toluene: ethyl acetate: acetic acid (7:2:1, v/v/v) for both the extracts. Plates were developed in a CAMAG twin-trough glass tank presaturated with mobile phase for 25 min at room temperature. Then, the plates were dried and scanned with a CAMAG TLC scanner (TLC scanner III) at 254 and 366 nm. The sample application, scanning, and interpretation were done using winCATS 1.2.3 software as per the previously reported protocol (Zahiruddin et al., 2020b).

For the chemical analysis of withanolide S in WSA and myristicin in MFA. The standards were dissolved in methanol (1 mg/ml). Thereafter, with the help of Camag Linomat-V (CAMAG, Switzerland), 2 μL of each samples (WS and MF) and standard (100–2000 ng) was applied with a 6 mm wide band length to Silica gel 60 F254 precoated TLC plates (10 cm × 10 cm; Merck, Germany) with the nitrogen flow providing a delivery speed of 120 nL/s. The plates were eluted to a distance of 8.0 cm at room temperature in the mobile phase toluene: ethyl acetate: acetic acid (5:4:1, v/v/v) for both the extracts. Plates were developed in a CAMAG twin-trough glass tank presaturated with mobile phase for 25 min at room temperature. After drying the spots on the developed plates, myristicin was visualized under visible 254 nm, and withanolide S was visualized after derivatization (5% anisaldehyde sulfuric acid) at 540 nm.

### TLC-Bioautography Screening of Anticholinesterase Active Metabolites

Further, enzyme inhibitory activities of the developed spots were detected by spraying the substrate, dye and enzyme (Elufioye et al., 2017). DTNB/ATCI reagent (1 mmol/L DTNB and 1 mmol/L ATCI in buffer A) was sprayed on the TLC plate until the silica on the plate was saturated with the solvent. It was allowed to dry for 5 min and then 3 U/mL of enzyme solution was sprayed onto the plate. White spots against yellow background indicated inhibiting compounds. The observed spots were recorded within 15 min so as to avoid disappearance of spots. The minimum concentration which could be visually detected was considered to be the detection limit. Retardation factor (R_f_) values and colour of the resolved bands were noted down.

### MS Analysis and Identification of Bioactive Metabolites Detected by TLC-Bioautography

The corresponding regions showing activity were scrapped off from the non-derivatized plates and eluted with methanol. The prepared solutions were filtered through 0.22 µM syringe filter for MS analysis. MS analysis was performed on Water’s ACQUITY UPLC system equipped with a binary solvent delivery system, an autosampler, column manager, and a tunable MS detector. The system was operated under the Empower software. Data acquisition has been done in negative modes. Chromatography was performed using methanol and 0.1%formic acid (8:2 v/v) as the mobile phase on monolithic capillary silica-based C18 column (ACQUITY UPLC BEH C18 1.7 µm, 2.1 mm × 100 mm), with the precolumn split ratio 1:5 and flow rate 10 μL/min at optimum temperature. Separation was achieved by stepwise gradients. The flow rate of the nebulizer gas was set to 500 L/h, for cone gas it was set to 50 L/h, and the source temperature was fixed to 100°C. The capillary and cone voltage were set to 3.0 and 40 KV, respectively. For collision, argon was used at a pressure of 5.3 × 10^−5^ Torr. The accurate mass and composition for the precursor ions and the fragment ions were calculated using the MassLynx V4.1 software incorporated in the instrument (Zahiruddin et al., 2017). Separated metabolites present in different samples were tentatively identified based on their m/z value from mass data bank and previously reported literature.

### *In vivo* Studies

#### Procurement, Housing and Acclimatization of Animals

12-week old male Wistar rats weighing 220 ± 20 g were selected for the experiment which was approved by the Institutional Animal Ethics Committee (IAEC) under the protocol number 1534. The animals were housed in the polypropylene cages (6 rats per cage) under the standard laboratory conditions of 12 hr light/12 hr dark cycle and controlled conditions of temperature and humidity (25 ± 2°C, 55–65%) with free access to standard rodent chow diet and water *ad libitum*. 2–3 days before the experiment, all the rats were acclimatized at the laboratory conditions in compliance with the guidelines of Committee for the Purpose of Control and Supervision on Experimentation on Animals (CPCSEA).

#### Drugs and Treatment Schedule

The rats were divided randomly into nine groups (*n* = 6 per group). For oral administration, the best active extracts of both the plants were dissolved in 0.5% carboxy methyl cellulose (CMC) solution. The rats in the control group i.e., Group 1 were given 0.5% CMC solution at a dose of 10ml/rat. Group 2 received scopolamine (2 mg/kg) for the induction of memory loss and served as negative control. Groups 3–6 treated with the high and low doses of WSA and MFA extracts respectively (BAE-I (high):7.6912 mg/rat; BAE-I (low):1.4498 mg/rat; BAE-II (low):0.7852 mg/rat; BAE-II (high):6.9652 mg/rat respectively in addition to scopolamine treatment. Whereas, Group 7 and 8 received the synergistic combinations in high and low doses of the selected BAEs (FD-I: BAE-I (high) + BAE-II (low); FD-II: BAE-I (low) + BAE-II (high)) respectively and served as test. Group 9 received piracetam (200 mg/kg) orally and served as positive control. The dose of the test samples (extracts) was calculated from the extractive value obtained with respect to the dose signified in Ayurvedic Pharmacopoeia. The drugs were administered for 30 days and on 30th, 31st, 32nd day, rats were subjected to spontaneous alternation behaviour (SAB) following which training on morris water maze was carried out for 5 days (from 33rd-37th day). Further dosing was continued from 30th-37th day. On 37th day, the reference memory test was performed in Morris water maze.

#### Elevated Plus Maze Test

Spontaneous alternation behaviour (SAB) was assessed by using four-arm wooden cross maze having all arms open (arms: length 23.5 cm, breadth 8 cm, wall height 10 cm) with a central platform (8 · 8 cm) was used and it was elevated at a height of 50 cm. This is unlike elevated plus maze where two arms are open and two are closed. Mice were placed on the central platform and allowed to traverse the maze freely for 6 min. The number and sequence of entries into the arms were recorded. An alternation was defined as entry into four different arms on overlapping quintuple sets. Five consecutive arm choices within the total set of arm choices made up a quintuple set (ABCDB was a quintuple while ABCCB was not as the entry in arm D is not included). Then percentage alternation was calculated as the ratio of actual alternation/possible alternation ×100. The possible alternation is equal to the total number of arm entries minus 4 (Vohora et al., 2005).

#### Morris Water Maze Test

Morris water maze was performed to assess the rat’s ability of learning navigatation to a specific location in a relatively large spatial environment. A round tank having 50 cm depth and 160 cm width having black lining was filled with water. The temperature was maintained at 20–24°C. An electronic computer having Tracking system SMART 3.0 (Panlab, Spain) was connected to the camera facing the tank in which experiment was to be performed to avoid manual tediousness of recording the swimming pattern of rats. The rats were allowed to rest in their cages and standard diet water *ad libitum* was made available during training sessions. The tank was divided into four equal quadrants (Target quadrant (T), Adjacent right (AR), Adjacent left (AL), and Opposite quadrant (O). An escape platform was submerged 2 cm beneath the surface of water in the target quadrant. The test was conducted for 5 days which included 4 days of training sessions and one day of probe trial. The rats were placed in the water tank facing the wall at one of the four quadrants (four starting points) during each trial. Before the training, rats were allowed to swim freely in the water for 120 s with the platform to adapt to the new environment. Each rat received four quadrants trials per day for three consecutive days, with an interval between each the trials of 15–20 min. The rats were given a lapse of 120 s (s) to locate the platform and were allowed to stay on the platform for 5s before being removed, however rats that were unable to find the platform within 120 s were placed on the platform for 10 s before being removed, and the escape latency was recorded using a video camera attached with the software. On the 37th day, the platform was removed and the number of entries over the previous platform location was noted and recorded over one trial of 120s. Tracking System SMART 3.0 (Panlab, Spain) software automatically recorded the time spent on the target quadrant (during probe trial) and the average distance covered while finding the platform.

## Results

The plant materials were authenticated as per the protocol described in Ayurveda and Unani pharmacopoeia. Further, voucher specimens of the same were deposited in the Herbarium of Bioactive Natural Product Laboratory, Jamia Hamdard, New Delhi, India for future reference (Table 1). The authenticated plant materials were extracted in two different solvents (Aqueous and hydroalcoholic) by 24 h maceration followed by reflux. The resultant extracts were filtered, evaporated to dryness, and the percentage yields were recorded (Table 2).

**TABLE 2:** Extractive values of selected plant extracts.

S.N.	Plant name	% Extractive yield (*n* = 3)	
		Aqueous extract (A)	Hydro alcoholic extract (HA)
1	*Bacopa monnieri*	7.82 ± 0.84	7.22 ± 0.72
2	*Piper nigrum*	6.58 ± 0.63	5.62 ± 0.69
3	*Withania somnifera*	10.62 ± 1.02	10.28 ± 1.24
4	*Centella asiatica*	16.67 ± 1.42	15.67 ± 1.53
5	*Nardostachys jatamansi*	8.23 ± 0.93	7.58 ± 0.91
6	*Myristica fragrans*	6.45 ± 0.62	7.96 ± 1.16
7	*Tinospora cordifolia*	7.62 ± 0.59	6.54 ± 0.71
8	*Butea monosperma*	7.28 ± 0.81	7.59 ± 0.83

### *In vitro* Anticholinesterase Inhibition Activity

**FIGURE 1:**
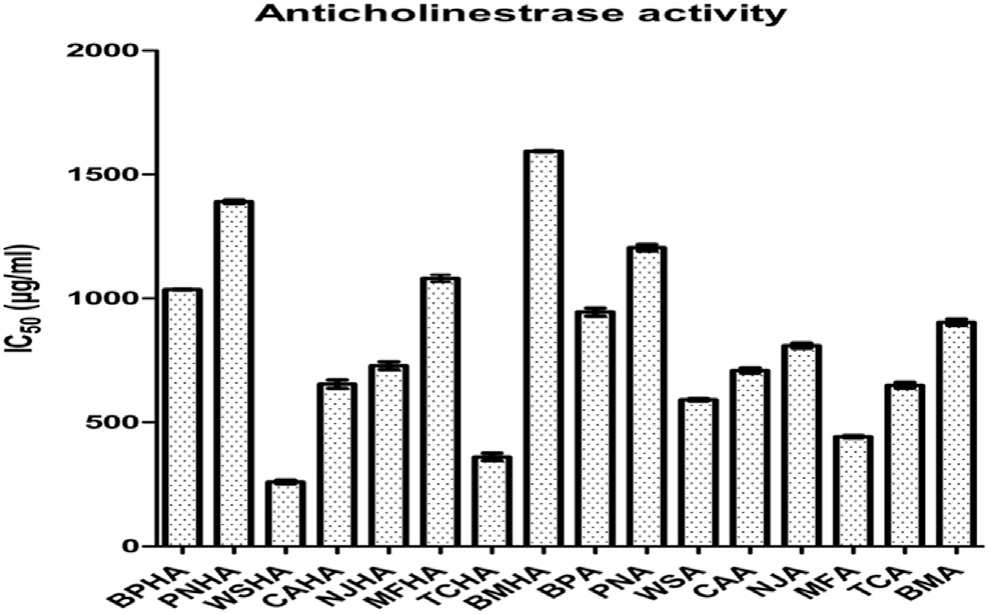
Anticholinesterase activity measured as IC50 value of selected plant extracts.

The highest 50% AChE enzyme inhibition was found in the *W. somnifera* (HA) followed by *T. cordifolia* (HA), *M. fragrans* (A), *W. somnifera* (A), *T. cordifolia* (A), *C. asiatica* (HA), *C. asiatica* (A), *N. jatamansi* (HA), *N. jatamansi* (A), *B. monosperma* (A), *B. monnieri* (A), *B. monnieri* (HA), *M. fragrans* (HA), *P. nigrum* (A), *P. nigrum* (HA) and *B. monosperma* (HA) (Figure 1 **)**.

Based on these observations two combinations of best active extracts were selected. The first combination included hydroalcoholic extracts of *W. somnifera* (WSHA) and *T. cordifolia* (TCHA)*,* which showed 50% inhibition against the enzyme AChE at a concentration of 266.19 ± 4.32 and 366.98 ± 5.02 (μg/ml) respectively and the second combination included extracts of *W. somnifera* (WSA) and *M. fragrans* (MFA), which showed 50% inhibition against the enzyme AChE at a concentration of 591.327 ± 5.32 and 441.917 ± 5.11 (μg/ml) respectively.

Further, both the combinations were repeated for inhibitory activity against the enzyme. The CI of WSHA and TCHA was found to be 0.477, whereas it was found to be 0.294 for WSA and MFA The calculations were based on constant ratio drug combination design. The CI values suggested that both the combinations exhibited a synergistic effect. The second combination was selected as the final candidate for further evaluation which included the extracts of WSA and MFA. Also, from the concentration vs. % inhibition plot, calculated CI values suggested that the combination of 1: 4.8 for WSA and MFA respectively, showed an inhibition of 64%. The same inhibition was also calculated at a ratio of 1: 9.72 for MFA and WSA respectively.

### Total Phenolic Content

The total phenolic content of MFA and WSA was found to be 12.33 ± 1.56 and 20.2 ± 2.34 mg GAE/g w/w of extracts respectively. These plant materials are the most abundant source of phenolic metabolites (Fernando et al., 2013; Gupta et al., 2013). The synergistic combination showed 25.28 ± 2.16 mg GAE/g w/w of combination extract. Phenolics are responsible for antioxidant activity. Larger the amount of phenols in extract, higher its antioxidant activity (Khan et al., 2017).

### Total Flavonoid Content

The total flavonoid content of MFA and WSA was found to be 5.81 ± 0.95 and 4.24 ± 1.19 mg RE/g w/w of extracts respectively, whereas the synergistic combination showed 8.42 ± 1.32 mg GAE/g w/w of combination extract. Hydroxyl groups present in flavonoids mediate their antioxidant potential by free radicals scavenging and metal ions chelating (Miguel et al., 2014).

### Antioxidant Potential

#### DPPH Scavenging Potential

This method is widely used to determine the free radical scavenging activities within a short time compared with other assays. The degree of discoloration showed the scavenging potential of an extract. The synergistic combination showed potent DPPH radical scavenging potential and it inhibited 81.65 ± 2.85% at 1,000 μg/ml, it was almost similar to a standard ascorbic acid at 500 μg/ml. The percentage inhibition of individual extract of WSA was shown 48.4 ± 1.85% at 1,000 μg/ml, whereas MFA at the same concentration exhibited 31.5 ± 2.17% at 1,000 μg/ml.

#### Reducing Power Potential

Antioxidant activity of synergistic combination as well as individual extract were measured by potassium ferricyanide (Fe^3+^) to potassium ferrocyanide (Fe^2+^) reduction method. An agent having a reduction potential undergoes reaction with Fe^3+^ to form Fe^2+^, and further reacts with ferric chloride to form ferric ferrous complex that has an absorption maximum at 700 nm. The synergistic combination showed potent DPPH radical scavenging potential and it inhibited 87.22 ± 2.84% at 1,000 μg/ml, it was almost similar to a standard ascorbic acid at 500 μg/ml. The individual extract results indicated that WSA has a higher reducing ability (55.22 ± 1.39% at 1,000 μg/ml) than MFA (43.22 ± 1.76% at 1,000 μg/ml).

### TLC Fingerprint and Chemical Analysis

TLC was used to separate the components of WSA and MFA. Different combinations of toluene, ethyl acetate and acetic acid were used as solvents to prepare the solvent system to achieve good separation of metabolites present in WSA and MFA. Separation of metabolites based on optimum bands with clear resolution in TLC plates were obtained using toluene: ethyl acetate: acetic acid in the ratio of 7:2:1 v/v/v.

**FIGURE 2:**
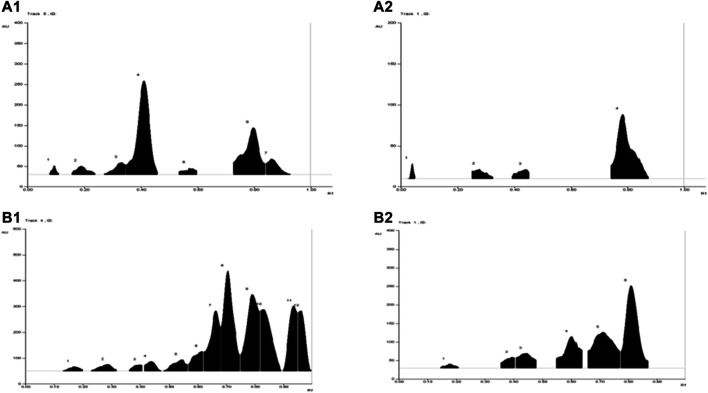
TLC chromatograms of WSA and MFA at different wavelengths. **(A1)** WSA at 254 nm **(A2)** WSA at 366 nm **(B1)** MFA at 254 nm **(B2)** MFA at 366 nm.

A total of seven metabolites were detected in WSA (R_f_ 0.09, 0.19, 0.33, 0.41, 0.57, 0.82, 0.91) when observed under UV 254 nm and four metabolites were detected (R_f_ 0.04, 0.28, 0.44, 0.74) under UV 366 nm**.** While in MFA 12 metabolites were detected under UV 254 nm (R_f_ 0.17, 0.29, 0.40, 0.44, 0.54, 0.61, 0.66, 0.71, 0.79, 0.82, 0.93, 0.96) and six metabolites were detected under UV 366 nm (R_f_ 0.17, 0.40, 0.44, 0.61, 0.71, 0.82). The same has been depicted in Table 3 and illustrated in Figure 2.

**TABLE 3:** TLC profiling of WSA and MFA separated in toluene: ethyl acetate: acetic acid (7:2:1, v/v/v) at 254 and 366 nm.

S.N.	Rf	WSA		MFA		
		(Wavelength)				
		254	366		254	366
1	0.04	−	+		−	−
2	0.09	+	−		−	−
3	0.17^a^ ^b^	−	−		+	+
4	0.19	+	−		−	−
5	0.28	−	+		+	−
6	0.33	+	−		−	−
7	0.40	+	−		+	+
8	0.44^a^ ^c^	−	+		+	+
9	0.54	−	−		+	−
10	0.57	+	−		−	−
11	0.61^a^ ^b^	−	−		+	+
12	0.66^a^ ^b^	−	−		+	−
13	0.71	−	−		+	+
14	0.74^a^ ^c^	−	+		−	−
15	0.79^a^ ^b^	−	−		+	−
16	0.82^a^ ^b^ ^c^	+	−		+	+
17	0.92^a^ ^c^	+	−		+	−
18	0.96	−	−		+	−

a Identified anticholinesterase active metabolites by TLC-MS-bioautography.

b Identified metabolite in MFA.

c Identified metabolite in WSA.

The average quantity of withanolide S in WSA and myristicin in MFA extract was found to be 5.11 ± 0.33% and 3.88 ± 0.41%, respectively. The TLC plate and their chromatograms are shown in Supplementary Figure S3.

### TLC-Bioautography Screening of Anticholinesterase Active Metabolites

**FIGURE 3:**
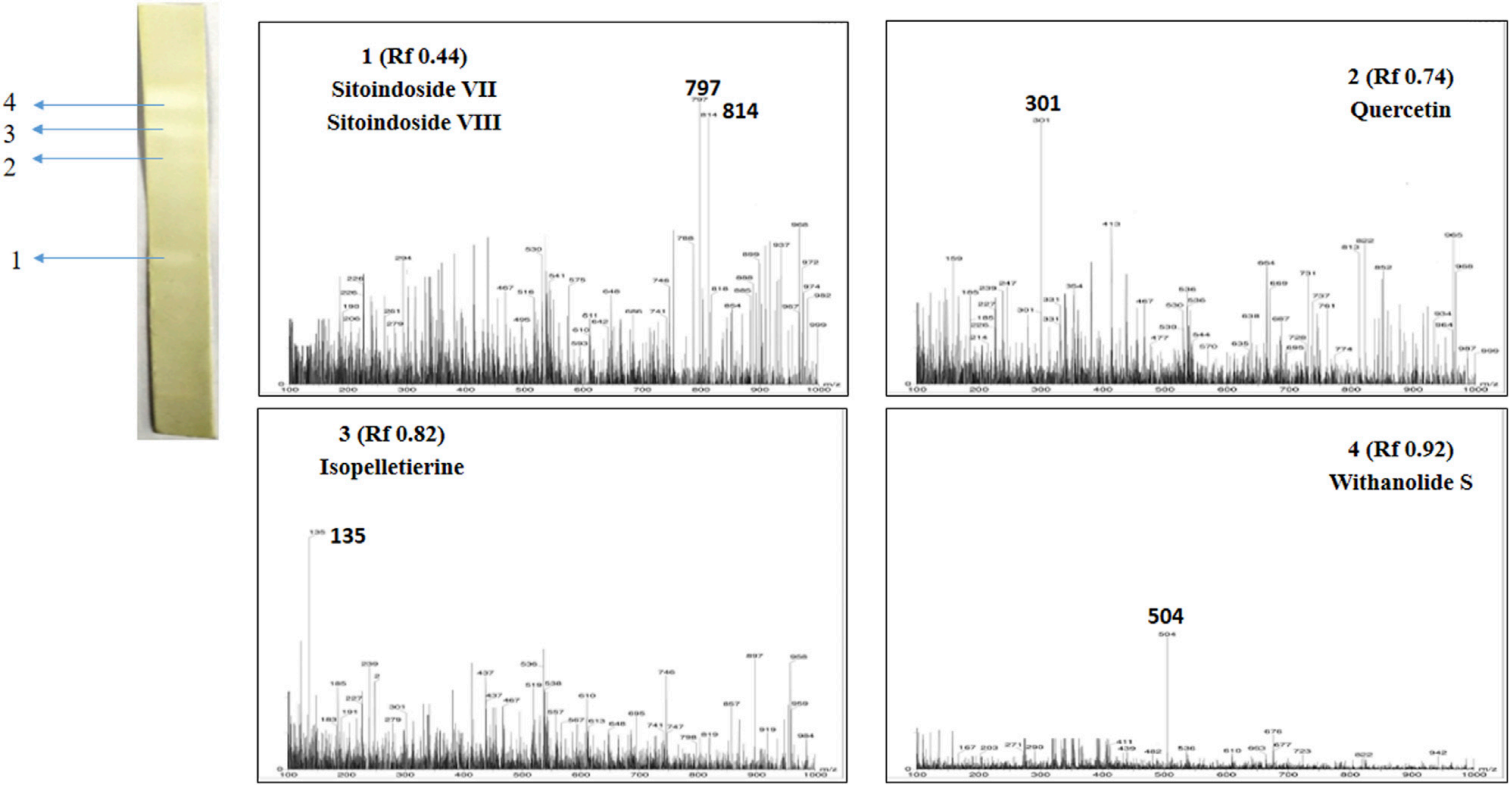
Developed TLC-bioautography plates of WSA represents AChE active spots and their mass spectroscopy.

**FIGURE 4:**
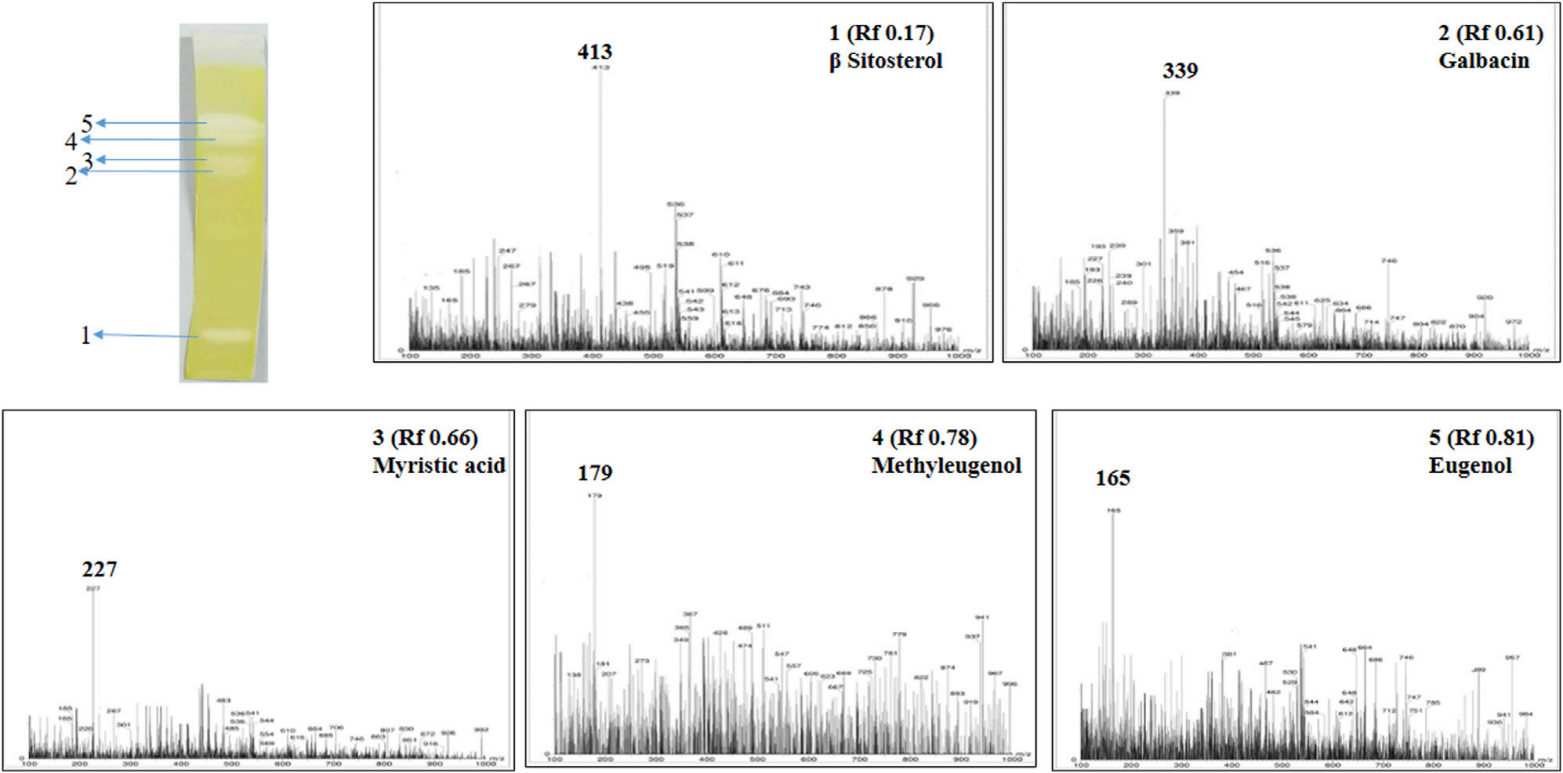
Developed TLC-bioautography plates of MFA represents AChE active spots and their mass spectroscopy.

Post TLC bioautography, WSA showed four white colored spots in the TLC plate (Figure 3 and Supplementary Figure S1) indicating acetyl cholinesterase inhibiting activity at R_f_ 0.44, 0.74, 0.82, 0.91. Whereas, MFA exhibited five white spots of anticholinesterase activity (Figure 4 and Supplementary Figure S2) at R_f_ 0.17, 0.61, 0.66, 0.78, and 0.81. The isolated bioactive metabolites from WSA and MFA are tabulated in Table 4.

**TABLE 4:** Isolated bioactive metabolites from WSA and MFA through TLC-MS-bioautography.

Extract	S.N.	R_f_ value	Tentative mass	Exact mass	Compound name	Chemical formula
WSA	1	0.44	797.0	798.0	Sitoindoside VII	C_50_H_86_O_7_
			814.0	814.0	Sitoindoside VIII	C_50_H_86_O_8_
	2	0.74	301.0	302.04	Quercetin	C_15_H_10_O_7_
	3	0.82	135.0	135.16	Isopelletierine	C_8_H_9_NO
	4	0.91	470.0	470.6	Withanolide S	C_28_H_40_O_8_
MFA	1	0.17	413.0	414.70	β Sitosterol	C_29_H_50_O
	2	0.61	339.0	340.37	Galbacin	C_20_H_20_O_5_
	3	0.66	227.0	228.20	Myristic acid	C_14_H_28_O_2_
	4	0.78	179.0	178.23	Methyleugenol	C_11_H_14_O_2_
	5	0.81	165.0	164.08	Eugenol	C_10_H_12_O_2_

### MS Analysis and Identification of Bioactive Metabolites Detected by TLC-Bioautography

Bioactive compounds detected on the TLC plates were isolated and directly subjected to MS analysis for its identification. The MS analysis was performed in both positive and negative ion mode. Detected bioactive compounds with potential anticholinesterase activity were identified by comparing experimental data for m/z of molecular ion peak and previously reported literatures. Total six compounds in WSA and five compounds in MFA were identified as compounds with anticholinesterase activity through TLC-bioautography-MS (Table 4).

In WSA, AChE inhibiting compounds identified were sitoindosides VII, VIII (R_f_ 0.44), quercetin (R_f_ 0.74), isopelletierine (R_f_ 0.82) and withanolide S (R_f_ 0.92) while in MFA, anticholinesterase compounds identified were β-sitosterol (R_f_ 0.17), galbacin (R_f_ 0.61), myristic acid (R_f_ 0.66), methyl eugenol (R_f_ 0.79), eugenol (R_f_ 0.82) and myristic acid (R_f_ 0.66) (Figures 3, 4). Structures of the identified constituents of MFA and WSA are depicted in Supplementary figure 4.

Considering this study, we can say that TLC-bioautography-MS would create a new hope in exploring lead compounds from natural products as well as establishing biological mechanisms of various extracts of natural products used in traditional systems of medicines. The data generated from TLC-bioautography-MS would be beneficial for the development of synergy based formulations and supports its acceptability.

### *In vivo* Nootropic Studies

Memory and learning ability test using Morris water maze model and Elevated plus-maze model was performed to examine the effect of best active extracts of WSA and MFA on albino Wistar rats individually and in combination.

The experimental rats were divided into nine groups and were subjected to training. The data were analyzed by one-way ANOVA followed by Dunnett’s multiple comparison test using Graph Pad prism software. The results displayed that there was a significant difference in the learning ability of group 2 rats (toxic control group) and the best active extracts (WSA and MFA) of the plants individually and in combination.

#### Effect of Morris Water Maze Training

**FIGURE 5:**
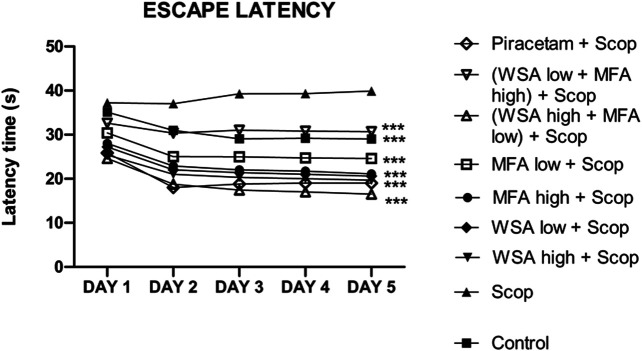
Dynamics of escape latency in the spatial acquisition trial in reference memory test in male wistar rats in Morris water maze. There were five days acquisition trials with 30 s inter-trial intervals and each data point is the mean of four trials. The data is represented as Mean ± SEM. Each line in the plot shows the average of six mice in each group. Markers represent the differences ***p < 0.001 when compared to Scop group; analyzed by one-way ANOVA followed by Dunnett’s Multiple Comparison Test.

During the acquisition sessions (days 33rd–36th) scopolamine treatment resulted in significantly longer escape latencies when compared to the control group (Figure 5). Significant decreases in escape latencies were observed when compared to the first day of the training sessions in the control group but not in the scopolamine treated rats.

The most significant decrease in escape latencies was seen in the synergistic combination group containing a higher concentration of WSA and lower concentration of MFA followed by a combination group with lower concentration of WSA. The escape latencies of the synergy group with higher concentration of WSA was comparable to that of the standard group.

**FIGURE 6:**
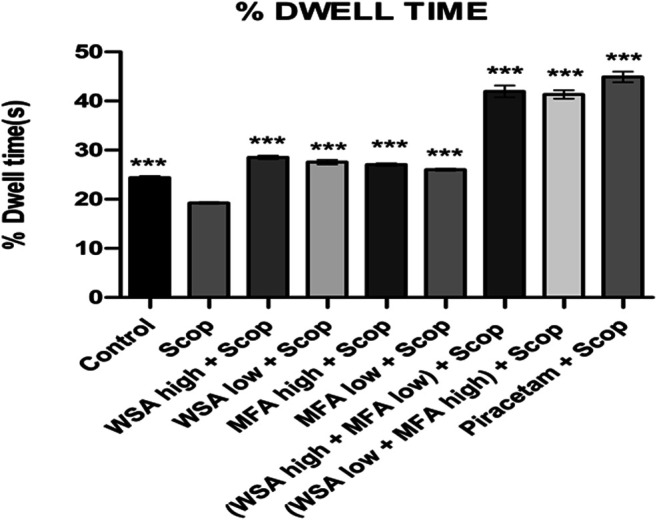
Dynamics of percentage dwell time in the spatial acquisition trial in reference memory test in male wistar rats in Morris water maze. Each data point is marked on the day of probe trial and is the mean of four trials. The data is represented as Mean ± SEM. Each line in the plot shows the average of six mice in each group. Markers represent the differences ***p < 0.001 when compared to Scop group; analyzed by one-way ANOVA followed by Dunnett’s Multiple Comparison Test.

In the probe trial session that is on day 37th, the time spent in the target quadrant (% dwell time) for combination groups was higher as compared to that of toxic group and the normal control during probe trial which validates the memory enhancement potential of the combination rats as depicted in Figure 6. The highest % dwell time was observed in the synergistic combination group containing a higher concentration of WSA and lower concentration of MFA followed by the combination group with lower concentration of WSA and higher concentration of MFA.

#### Elevated Plus Maze Performance

**FIGURE 7:**
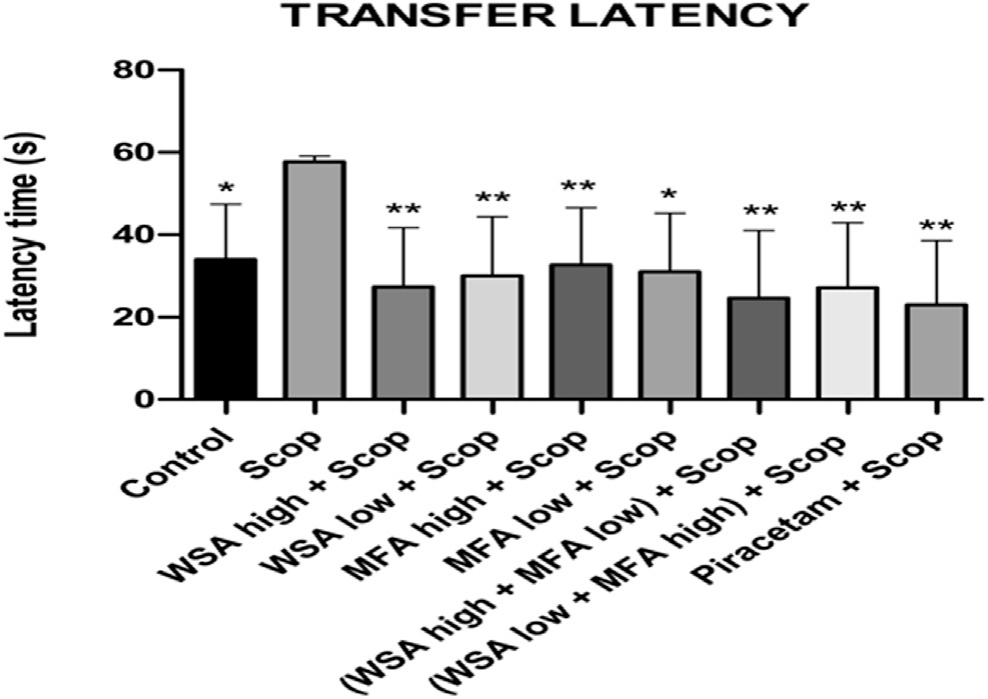
Dynamics of transfer latency in the spatial acquisition trial in reference memory test in male wistar rats in Elevated plus maze. There were three days acquisition trials with 30s inter-trial intervals and each data point is the mean of four trials. The data is represented as Mean ± SEM. Markers represent the differences. Each line in the plot shows the average of six mice in each group. The data is represented as Mean ± SEM. Markers represent the differences **p < 0.001 and *p < 0.5 when compared to Scop group; analyzed by one-way ANOVA followed by Dunnett’s Multiple Comparison Test.

During the acquisition sessions (days 30th–32nd) scopolamine treatment resulted in significantly longer transfer latencies when compared to the control group (Figure 7). Significant decreases in transfer latencies were observed when compared to the first day of the training sessions in the control group but not in the scopolamine treated rats.

The most significant decrease in latencies was noted in the synergistic combination group containing a higher concentration of WSA and lower concentration of MFA followed by a combination group with lower concentration of WSA. However, the latencies of the synergy group with higher concentration of WSA were comparable to that of the standard group.

## Discussion

The present study helped in screening out the best active extracts of AChE inhibitors from AYUSH based medicinal plants (*B. monnieri, P. nigrum, W. somnifera, C. asiatica, N. jatamansi, M. fragrans, T. cordifolia and B. monosperma*) reported for learning and memory enhancement. After extraction with aqueous and hydroalcoholic solvent, all these extracts were screened for their *in vitro* AChE activity. Based on their *in vitro* AChE activity, WSA and MFA were selected for development of synergistic combination. Surprisingly, our data showed that the aqueous extracts of the selected plants showed synergism. In the traditional system of medicine, aqueous extract is more preferable as compared to others because of being comparatively safer (Khan et al., 2017). The active combination of WSA and MFA showed a good synergistic effect when given in combination according to constant ratio combination design. It decreased inhibitory concentration by 6.6 and 7.4 folds for WSA and MFA, combination. The selected plants are previously reported to be beneficial in a variety of brain disorders (Parle et al., 2004; Mukherjee et al., 2007).

Quality control profile is required to the regulatory bodies for plant based medicinal products (Calixto, 2000). Many reports of WS and MF are published for their AChE activity without having any analytical study. Quality control analysis of WS and MF showing AChE activity has been carried out through TLC profiling. This TLC profiling and chemical analysis can be used as quality control parameters of the extract with its AChE activity.

The combination extracts showed anticholinesterase activity and then the activity was traced back to the bioactive metabolites present in the aqueous extracts of selected combination of two plants i.e., WSA and MFA by TLC-bioautography coupled with MS. In WSA, sitoindosides VII, VIII, quercetin, isopelletierine and withanolide S were identified as AChE inhibiting compounds. Most of the identified compounds in WSA were previously reported for their AChE inhibiting activity (Narinderpal et al., 2013; Ademosun et al., 2016). While in MFA eugenol, methyl eugenol, myristic acid, galbacin and β-sitosterol were identified as an AChE inhibiting compounds. These identified compounds showed potent AChE inhibitory activity (Cuong et al., 2014; Mukherjee et al., 2014; Ayaz et al., 2017; Vargas-Méndez et al., 2019). TLC-MS-bioautography is a simple and fast method to identify bioactive compounds showing AChE activity.

The synergistic combination as well as the individual extracts have been estimated for their antioxidant activity by two methods because antioxidant activity happens through various mechanisms and is influenced by various reasons, which cannot be fully defined by one method. The synergistic combination has more antioxidant potential as compared to WSA and MFA extracts, as well as the phenolics and flavonoid contents in synergistic combination showed better result as compared to extracts and these polyphenolics may be responsible for their antioxidants activity (Geetha et al., 2003). The results from DPPH and reducing power assay suggested that besides inhibiting AChE, the combination also acts as a potent antioxidant which further aids as a complementary mechanism to counter amnesia (Khan et al., 2017).

From a total of 16 extracts from eight plants, the active combination of WSA and MFA showed a good synergistic effect when given in combination according to constant ratio combination design. It decreased inhibitory concentration by 6.6 and 7.4 folds for WSA and MFA, combination.

Morris water maze test has been used in various experiments to assess the effect of drugs on cognitive impairment (Webster et al., 2014; Xu et al., 2019; Schröder et al., 2020) Hence, it was performed to validate the potential of WSA and MFA in management of Alzheimer’s disease. However, various types of water maze models are available, Morris water maze model possess additional advantages (Bromley-Brits et al., 2011). Elevated plus maze model has been also used to determine the effect of various drugs on memory and learning (Jürgenson et al., 2010). Results suggested that both the synergy based combinations effectively increased learning and acquisition (Morris Maze) along with enhancing reference memory (elevated plus maze). The given doses of extracts were in compliance with Ayurvedic Pharmacopoeia and were administered for 30 days orally (individually and in combination). Scopolamine given through the intraperitoneal route induced significant memory loss and served as a negative control (Adabizadeh et al., 2019). On the other hand, Piracetam, a standard cholinergic drug was used as a positive control (Leuner et al., 2010). The analysis was done by one-way ANOVA method followed by Dunnett’s multiple comparison test using Graph Pad prism software which helped in doing an efficient comparison of the individual and combination extracts with the toxic group. Further, low % dwell time along with high transfer latencies and escape latencies in the scopolamine treated rats compared to the control and other groups suggested that scopolamine effectively reduced learning and memory in rats. High % dwell time signifies that the treated subjects were able to memorize the target quadrant area for which they were being trained and spent sufficiently more time in that particular area. Whereas, the transfer latencies i.e. escape latency (Morris maze) and Transfer latency (Elevated plus maze) signifies that the time taken to locate the platform was remarkably reduced in comparison to the toxic group which showed improved learning. Also, from the two combinations, the one with the higher dose of WSA showed the best learning and memory enhancement.

## Conclusion

The present study followed an exhaustive approach in screening and selecting the best active synergistic combination of extracts of nootropic plants with anticholinesterase activity. The bioactive anticholinesterase metabolites were detected and identified and served as a strong evidence of the potential of this combination. Further, results from the *in vitro* studies of DPPH and reducing power assay suggested that the combination provides strong antioxidant effect and thus ameliorates neuronal degeneration. The *in vivo* studies suggested that combination possessed excellent learning and memory enhancing effects in rats. The present approach provides strong evidence for the acceptability of the developed synergistic combination for learning and memory enhancement. Further, molecular studies need to explore the mechanism behind the learning and memory enhancing effect of the synergistic combination.

## Data Availability

The raw data supporting the conclusion of this article will be made available by the authors, without undue reservation, to any qualified researcher.

## Abbreviations

BAE, Best active extract; CMC, Carboxy methyl cellulose; CPCSEA, Control and supervision on experimentation on animals; FD, Fixed dose; IAEC, Institutional animal ethics committee; MF, *Myristica fragrans*; MFA, *Myristica fragrans* aqueous; nL; RE/g, Rutin equivalent per Gram; Rf, Retention factor; Rpm, Rotation per minute; SAB, Spontaneous alternation behaviour; SAB, Spontaneous alternation behaviour; TCHA, *Tinospora cordifolia* hydroalcoholic; U, units; UPLC, Ultra performance liquid chromatography; UV, Ultra violet; v/v, Volume by volume; WS, *Withania somnifera*; WSA, *Withania somnifera* aqueous; WSHA, *Withania somnifera* hydroalcoholic.
