# Supplementary material for: Development of Synergy-Based Combination for Learning and Memory Using in vitro, in vivo and TLC-MS-Bioautographic Studies
Source: Front Pharmacol. 2021 Jul 2;12:678611. doi: 10.3389/fphar.2021.678611 (PMC8283279; doi:10.3389/fphar.2021.678611)
Supplement: Supplementary file 1 [file Presentation1.PPTX]

## Slide 1
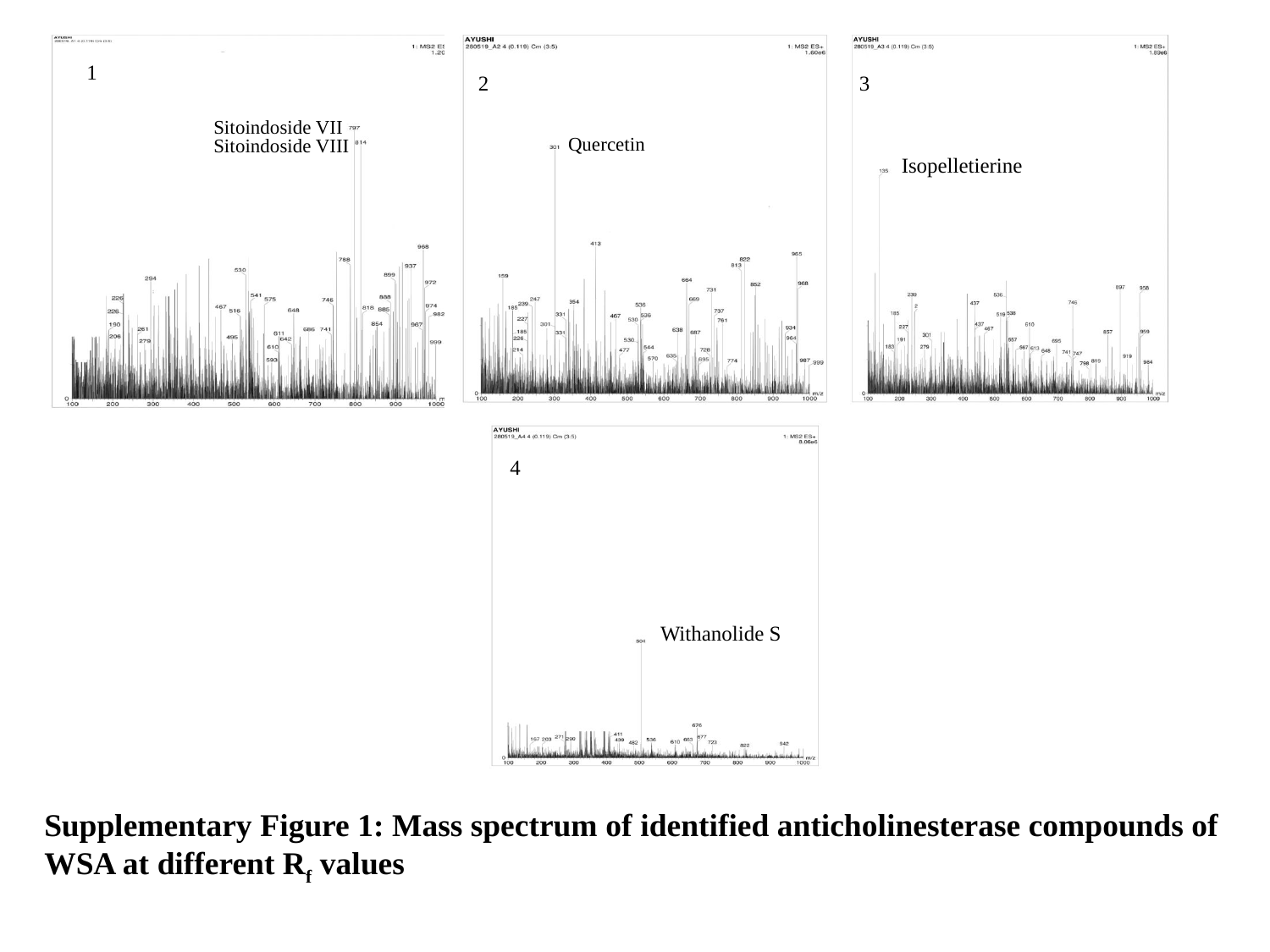

1
2
3
Sitoindoside VII
Quercetin
Sitoindoside VIII
Isopelletierine
4
Withanolide S
Supplementary Figure 1: Mass spectrum of identified anticholinesterase compounds of WSA at different Rf values

## Slide 2
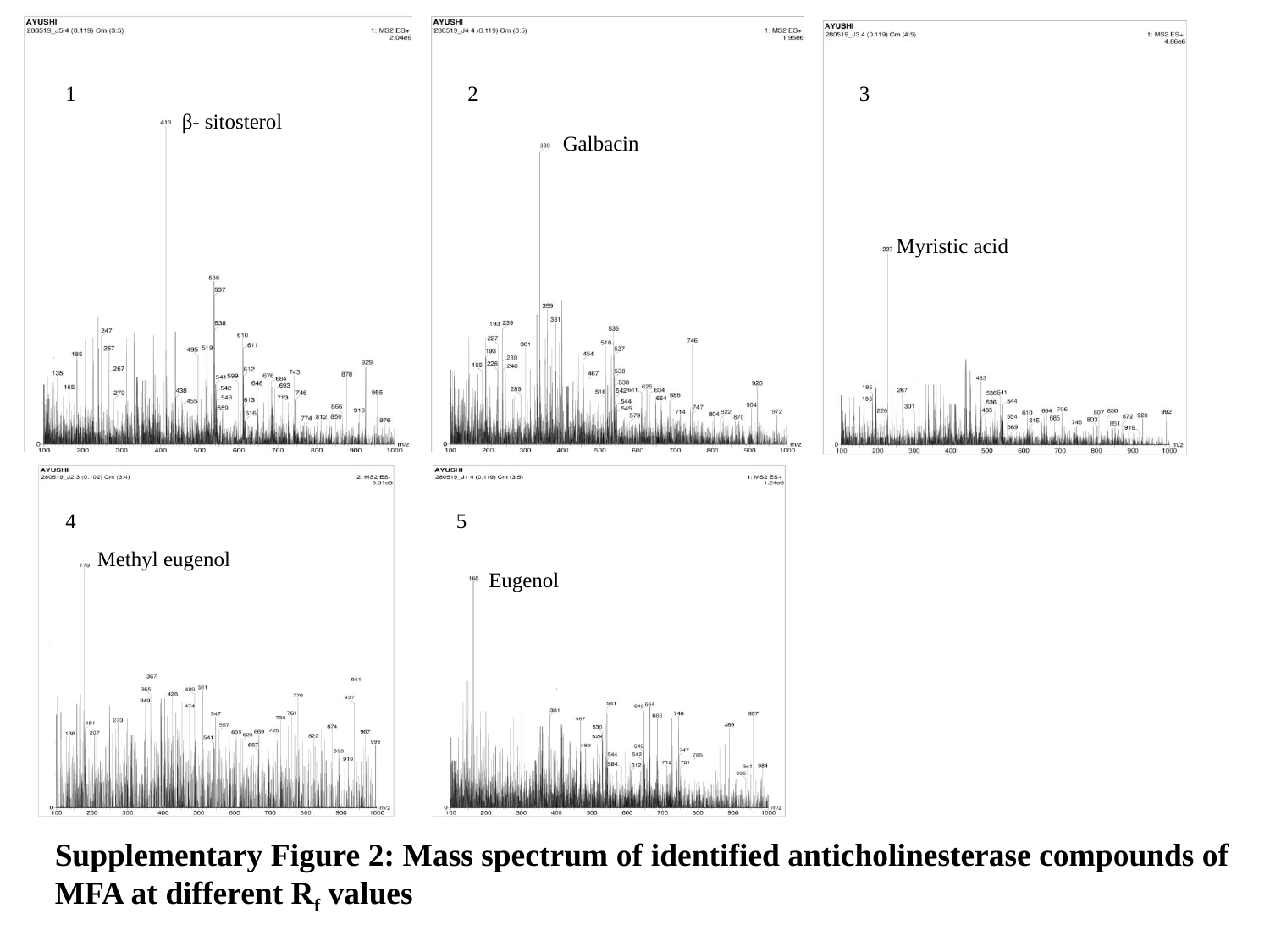

3
1
2
β- sitosterol
Galbacin
Myristic acid
5
4
Methyl eugenol
Eugenol
Supplementary Figure 2: Mass spectrum of identified anticholinesterase compounds of MFA at different Rf values

## Slide 3
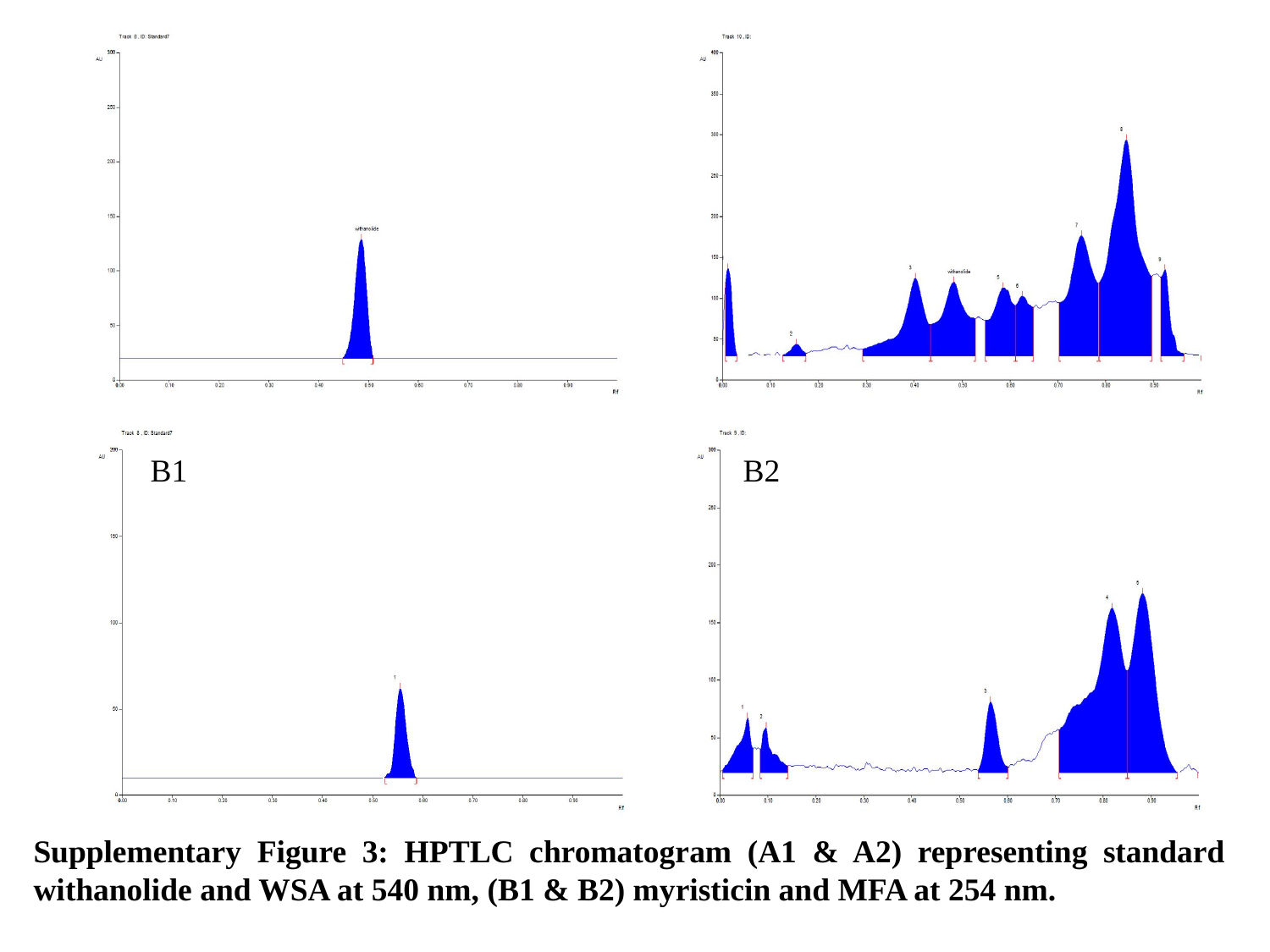

B1
A1
A2
B2
Supplementary Figure 3: HPTLC chromatogram (A1 & A2) representing standard withanolide and WSA at 540 nm, (B1 & B2) myristicin and MFA at 254 nm.
