# Supplementary material for: Development of Synergy-Based Combination for Learning and Memory Using in vitro, in vivo and TLC-MS-Bioautographic Studies
Source: Front Pharmacol. 2021 Jul 2;12:678611. doi: 10.3389/fphar.2021.678611 (PMC8283279; doi:10.3389/fphar.2021.678611)
Supplement: Supplementary file 2 [file Presentation2.PPTX]

## Slide 1
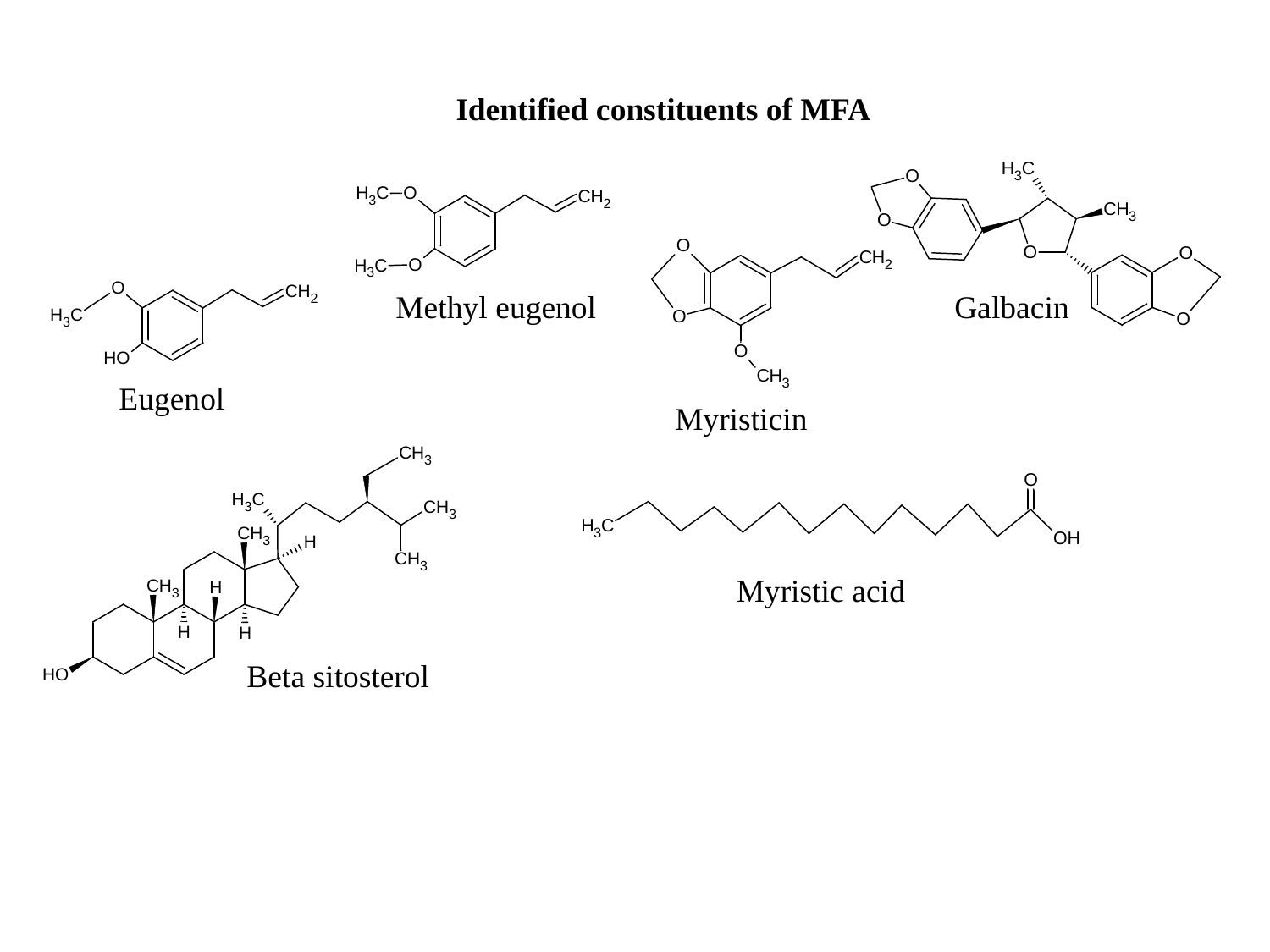

Identified constituents of MFA
Methyl eugenol
Galbacin
Eugenol
Myristicin
Myristic acid
Beta sitosterol

## Slide 2
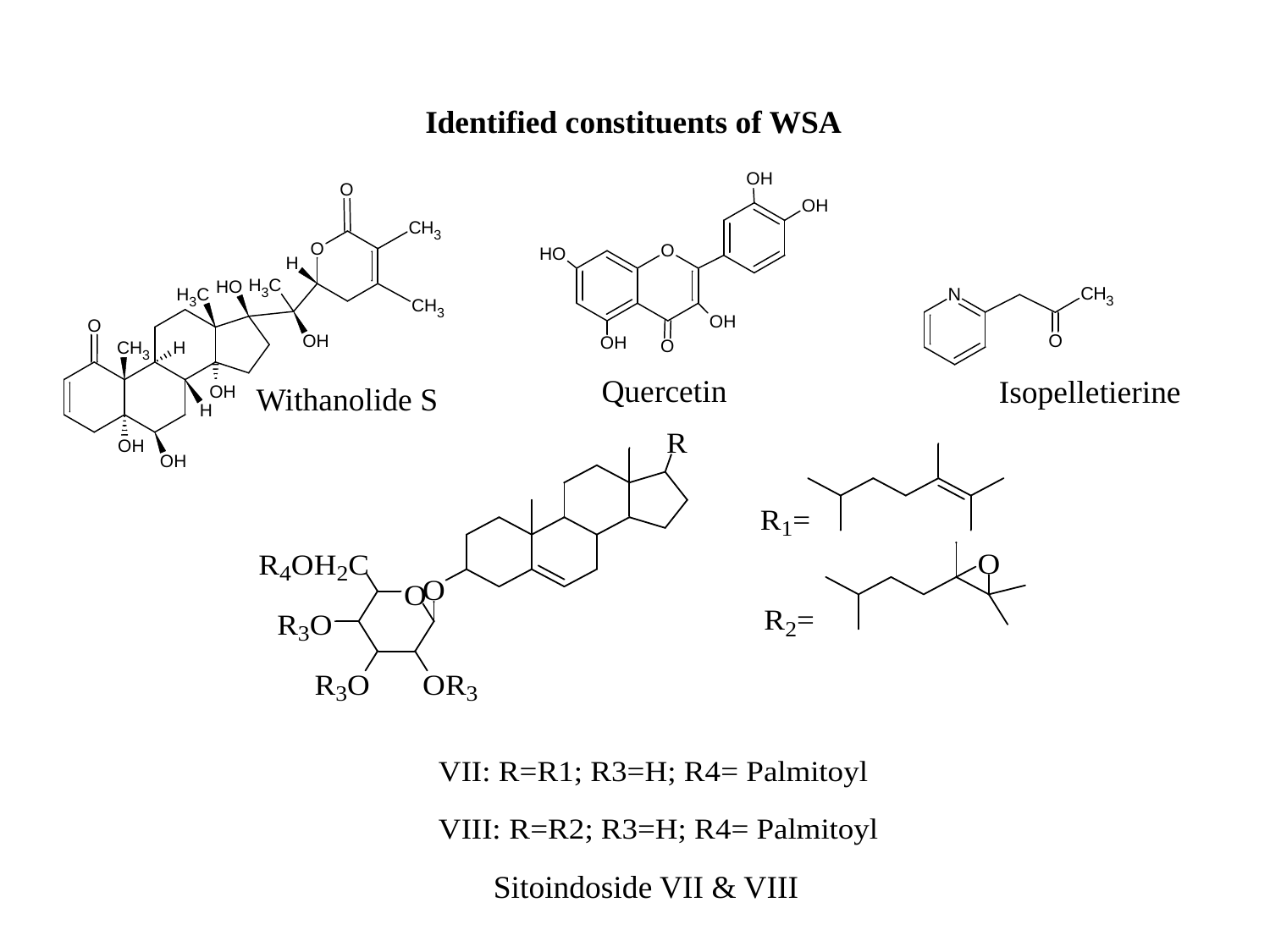

Identified constituents of WSA
Quercetin
Isopelletierine
Withanolide S
Sitoindoside VII & VIII
